# Comparison of prokaryotic community structure from Mediterranean and Atlantic saltern concentrator ponds by a metagenomic approach

**DOI:** 10.3389/fmicb.2014.00196

**Published:** 2014-05-08

**Authors:** Ana B. Fernández, Blanca Vera-Gargallo, Cristina Sánchez-Porro, Rohit Ghai, R. Thane Papke, Francisco Rodriguez-Valera, Antonio Ventosa

**Affiliations:** ^1^Department of Microbiology and Parasitology, Faculty of Pharmacy, University of SevillaSevilla, Spain; ^2^Evolutionary Genomics Group, Departamento de Producción Vegetal y Microbiología, Universidad Miguel Hernández, San Juan de AlicanteAlicante, Spain; ^3^Department of Molecular and Cell Biology, University of ConnecticutStorrs, CT, USA

**Keywords:** metagenomics, haloarchaea, halophilic bacteria, saltern, prokaryotic diversity

## Abstract

We analyzed the prokaryotic community structure of a saltern pond with 21% total salts located in Isla Cristina, Huelva, Southwest Spain, close to the Atlantic ocean coast. For this purpose, we constructed a metagenome (designated as IC21) obtained by pyrosequencing consisting of 486 Mb with an average read length of 397 bp and compared it with other metagenomic datasets obtained from ponds with 19, 33, and 37% total salts acquired from Santa Pola marine saltern, located in Alicante, East Spain, on the Mediterranean coast. Although the salinity in IC21 is closer to the pond with 19% total salts from Santa Pola saltern (designated as SS19), IC21 is more similar at higher taxonomic levels to the pond with 33% total salts from Santa Pola saltern (designated as SS33), since both are predominated by the phylum *Euryarchaeota*. However, there are significant differences at lower taxonomic levels where most sequences were related to the genus *Halorubrum* in IC21 and to *Haloquadratum* in SS33. Within the *Bacteroidetes*, the genus *Psychroflexus* is the most abundant in IC21 while *Salinibacter* dominates in SS33. Sequences related to bacteriorhodopsins and halorhodopsins correlate with the abundance of *Haloquadratum* in Santa Pola SS19 to SS33 and of *Halorubrum* in Isla Cristina IC21 dataset, respectively. Differences in composition might be attributed to local ecological conditions since IC21 showed a decrease in the number of sequences related to the synthesis of compatible solutes and in the utilization of phosphonate.

## Introduction

Hypersaline habitats are characterized by high salt concentrations, in addition to other features, such as high or low temperatures, high pH, and/or low oxygen concentrations (Javor, 1989; Rodríguez-Valera, 1993). Hypersaline environments are often aquatic systems (thalassohaline, of marine origin, or athalassohaline, formed by dissolution of mineral salt deposits of continental origin) or saline soils (Walsh et al., 2005; Ventosa, 2006; Ventosa et al., 2008), but they are also represented by salt deposits, some desert plants, oilfield brines and a variety of salted foods, from seasoned fish or meat to fermented foods as well as animal hides (Grant et al., 1998; Ventosa, 2006). The best studied hypersaline habitats are aquatic hypersaline systems, such as salt lakes and salterns.

Salterns are excellent models for studying the ecology and diversity of microorganisms, given that they are composed by a series of ponds with widely different salinities that concentrate salt from seawater to the point of saturation and precipitation. Most saltern studies have been performed on the saturated brine crystallizer ponds (Antón et al., 1999, 2000; Benlloch et al., 2001; Pašić et al., 2005; Pasić et al., 2007; Legault et al., 2006; Oh et al., 2010). Some comparative reports performed on crystallizers ponds, as Pasić et al. (2007), compared haloarchaeal communities from two Adriatic solar salterns, showing differences in the microbiota and that climate could play a role in the microbial community structure. Oh et al. (2010) examinated the diversity of *Haloquadratum* and other haloarchaea in three coastal, but geographically distant saltern crystallizer ponds in Australia. The great majority of the 16S rRNA gene sequences recovered from these crystallizers were related to *H. walsbyi* and diverged by less than 2% from each other, and from the type strain of this genus (strain C23). However, our knowledge about intermediate salinity ponds is limited. Benlloch et al. (2002) analyzed 16S rRNA sequences by DGGE of three salt ponds (8, 22, and 32% total salts, respectively) from Santa Pola saltern in Eastern Spain. Most bacterial sequences in the 8% salt pond were related to organisms of marine origin belonging to representatives of the classes *Alpha*-, *Beta*-, *Gamma*-, and *Epsilon*-*Proteobacteria*, and the phyla *Bacteroidetes*, *Actinobacteria*, and *Cyanobacteria*. In the 22% salt pond were found *Alpha*- and *Gamma*-*Proteobacteria*, *Cyanobacteria*, and *Bacteroidetes*, and most of them were related to specialized halophiles. From the 32% salt pond, the only *Bacteria* found were sequences that clustered with *Salinibacter ruber*, an extremely halophilic *Bacteroidetes*. And in those three different salinity ponds, most of the clones were related to cultured strains of the archaeal class *Halobacteria*. A metagenomic study of an intermediate salinity pond from Santa Pola saltern (SS19), revealed a low presence of bacterial species of *Halomonas*, *Chromohalobacter*, or *Salinivibrio* (Ghai et al., 2011), which are commonly isolated from those habitats (Arahal et al., 2001; Arenas et al., 2009) but abundant metagenomic reads affiliated to *Haloquadratum walsbyi* and *Salinibacter ruber* were also found. A novel representative of nanohaloarchaeota (“*Candidatus* Haloredivivus”) which was also found to be abundant, and its genome was partially assembled. The most abundant bacterium found in this 19% salt pond appeared to be a gammaproteobacterium closely related to *Alkalilimnicola* and *Nitrococcus*. This microbe has been recently cultured and its genome sequenced (Leon et al., 2013; López-Pérez et al., 2013). Besides, Ghai et al. (2011) found a large number of sequences related to presumably non-halophilic bacterial genera and, a group of low G+C *Actinobacteria* typical to freshwater habitats. A recent metagenomic study on a pond with 13% salts of Santa Pola saltern showed a large microbial diversity representing seven major higher taxa: *Euryarchaeota, Gammaproteobacteria, Alphaproteobacteria, Actinobacteria, Bacteroidetes, Verrucomicrobia*, and *Betaproteobacteria* (Fernández et al., 2014a). Community analysis of an intermediate salinity pond (18% total salts) from a saltern in Guerrero Negro, Mexico, demonstrated that the archaeal community was dominated by a single uncultured 16S rRNA phylotype with 99% similarity to sequences recovered from a Tunisian saltern and the most abundant bacterial sequences were 99% similar to an uncultured gammaproteobacterial clone from the Salton Sea (Dillon et al., 2013). Boujelben et al. (2012) explored the prokaryotic community in several ponds from Sfax saltern in Tunisia. They showed that some phylotypes, such as those related to *Haloquadratum* or representatives of *Bacteroidetes*, displayed a strong dependence of salinity and/or magnesium concentrations and that temperature was a strong factor structuring the prokaryotic community in the pond with 20% salinity, but not in the crystallizer pond, due to the seasonal changes. However, a survey about six salt lakes in Inner Mongolia, China, and a salt lake in Argentina showed that archaeal biogeography was influenced by Na^+^, CO^2−^_3_, HCO^3−^, pH and temperature, and bacterial biogeography was influenced by Na^+^, Mg^2+^, HCO^3−^, and pH as well as geographic distance (Pagaling et al., 2009; Grant et al., 2011).

Therefore, in order to learn more about the community structure of intermediate salinity ponds, we explored the phylogenetic and taxonomic differences as well as metabolic profiles of two geographically distant habitats from Spain: the Isla Cristina saltern that gets its water from the Atlantic Ocean and the Santa Pola saltern on the Mediterranean Sea.

## Materials and methods

### Sample collection, DNA extraction, and sequencing

The metagenomic datasets analyzed in this study are derived from different saline systems: one dataset was obtained from a pond at the Isla Cristina saltern located on the Atlantic Ocean, in southwestern Spain with a salinity of 21% (Fernández et al., 2014b); four datasets were from the Santa Pola saltern located in eastern Spain on the Mediterranean Sea, of which three were from concentrator ponds (SS13, SS19, and SS33) and one from a crystallizer pond (SS37) with salt concentrations of 13, 19, 33, and 37%, respectively (Ghai et al., 2011; Fernández et al., 2013, 2014a); and two marine datasets, deep chlorophyll maximum from Mediterranean Sea (DCM3) and Mar Menor coastal lagoon (MM5) with salinities of 3.8 and 5%, respectively (Ghai et al., 2010, 2012). All databases were obtained using the same DNA extraction method and the samples were sequenced by pyrosequencing 454 (Martín-Cuadrado et al., 2007; Ghai et al., 2011). The accession numbers for the deposited databases are shown in Table 1.

**Table 1:** **Features of the different datasets from saline habitats used in this study**.

**Datasets**	**Salinity (%)**	**Temperature (°C)**	**pH**	**Number of reads**	**Dataset size (Mb)**	**Average read length (bp)**	**Accession number/source**	**References**
Deep Chlorophyll Maximum (DCM3)	3.8	15.9	8.1	1,204,321	312	259	SRP002017	Ghai et al., 2010
Mar Menor Coastal Lagoon (MM5)	5	19	8.4	730,997	243	335	http://camera.calit2.net	Ghai et al., 2012
Santa Pola Saltern (SS13)	13	29	8.0	1,443,593	441	305	SRP028290	Fernández et al., 2013, 2014a
Santa Pola Saltern (SS19)	19	30	8.0	1,315,302	475	361	SRP007685	Ghai et al., 2011
Santa Pola Saltern (SS33)	33	30	7.0	842,872	309	367	SRP028290	Fernández et al., 2013
Santa Pola Saltern (SS37)	37	41	7.1	760,740	309	417	SRP007685	Ghai et al., 2011
Isla Cristina Saltern (IC21)	21	25	7.5	1,223,923	486	397	SRP029970	Fernández et al., 2014b

### Comparative analysis of metagenomic reads

To estimate cumulative nucleotide differences between metagenomic datasets, we carried out BLASTN searches of the complete set of sequences from every dataset vs. all the others. Bitscores of the top high-scoring segment pairs (HSPs) from every sequence from one set vs. another were summed to yield a cumulative pairwise bitscore value (CPBV) that was normalized and used to construct a distance matrix. CPBVs were normalized by dividing each one by the cumulative bistscore value derived from the BLASTN of one dataset vs. itself. The distance matrix was analyzed using the Phylip package (Felsenstein, 1989) to obtain a neighbor-joining tree.

G+C contents were computed using the program geecee in the EMBOSS package (Rice et al., 2000) and the amino acid frequency was calculated from a perl script. The metagenomic reads were annotated using UniProtKB database released in December 2013 (UniProt Consortium, 2014) through BLASTX search with a cutoff *e*-value of 1e-5.

16S ribosomal RNA genes were identified by comparing the datasets against the RDP database version 11.1 (Cole et al., 2014). All reads that matched a 16S rRNA sequence with an alignment length of more than 100 bp and an *e*-value lower than 1e-5 against the database were extracted. The best hit that was not described as unknown or unidentified was considered a reasonable closest attempt for classifying the 16S rRNA sequences. Sequences were assigned to a specific genus if they shared ≥95% 16S rRNA sequence identity with a known representative.

### Metagenomic reads assembly

Assembly of the metagenomic reads with greater than 100 bp was performed using stringent criteria requiring an overlap of at least 80 bp, 99% identity and at most a single gap in the alignment (using Geneious Pro 5.4). Next, assembled contigs that were less than 3 kb in length, and those with less than three predicted genes were discarded for further analysis. We retained only those contigs that provided consistent query hits to only single high level taxa (e.g., *Alphaproteobacteria*, *Euryarchaeota*, *Bacteroidetes*, *Actinobacteria*). To test if the assembly strategy produced authentic contigs from known organisms, we manually identified all contigs that belonged to *H. walsbyi*, one of the abundant organism in the datasets. The criterion was that all genes from a putative *H. walsbyi* contig must return best hits from that genome.

Tetranucleotide frequencies of the assembled contigs were computed using the wordfreq program in the EMBOSS package (Rice et al., 2000), and principal component analysis (PCA) was performed using the R package FactoMineR (Lê et al., 2008).

### Construction of phylogenetic trees

Maximum likelihood reference trees were constructed using RaxML as implemented in ARB software package (Ludwig et al., 2004) using reference 16S rRNA gene sequences with near full length (>1300 nt) from cultured isolates. Later, partial 16S rRNA gene assembled metagenomic sequences and closely related environmental uncultured 16S rRNA gene sequences were inserted into reference trees without altering tree topology using maximum parsimony criterion and a 50% base frequency filter. Bootstrap values greater than 50% are indicated above nodes and the scale bar represents 10 base substitutions per 100 nt positions. The 16S rRNA gene sequences retrieved in this study were deposited in Genbank under accession numbers KJ546108–KJ546118, KJ588879–KJ588888, KJ588892–KJ588898 (archaeal) and KJ588890–KJ588891, KJ588899–KJ588905 (bacterial).

## Results and discussion

### Features of datasets

**Figure 1:**
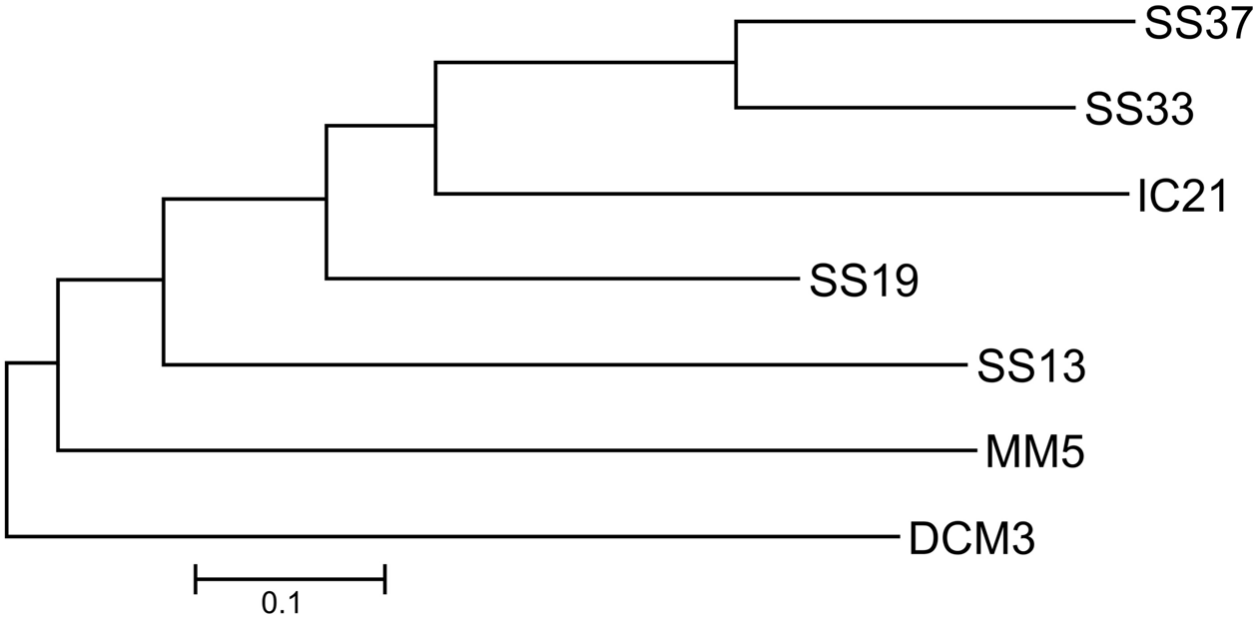
**Unrooted neighbor-joining phylogenetic tree based on a distance matrix calculated using “bit-scores” between the different metagenomes**. DCM3: Deep Chlorophyll Maximum, Mediterranean Sea (3.8% salinity), MM5: Mar Menor Coastal Lagoon (5% salinity), IC21: Isla Cristina saltern (21% salinity), SS13: Santa Pola saltern (13% salinity), SS19: Santa Pola saltern (19% salinity), SS33: Santa Pola saltern (33% salinity), and SS37: Santa Pola saltern (37% salinity).

In this study several metagenomic datasets were examined (Table 1). The thalassosaline waters of the salterns in Santa Pola and Isla Cristina have a marine origin but they have a different source: the Mediterranean Sea and the Atlantic Ocean, respectively. Besides their high salt concentrations, these environments are subject to strong solar irradiation (Rodríguez-Valera et al., 1985; Rodríguez-Valera, 1988). In order to determine how a gradient of salinity influences microbial communities, we compared metagenomes datasets of Santa Pola and Isla Cristina salterns and DCM3 and MM5 from marine sites (Figure 1). We expected marine derived datasets to form their own branch separate from the saltern datasets and that IC21 should be closer to SS19 than SS33, because salinity has been identified as the main factor determining the distribution of prokaryotic organisms in aquatic systems (Lozupone and Knight, 2007; Schapira et al., 2009). As expected, the phylogenetic tree showed that marine datasets, DCM3 and MM5 clearly differed from the saltern datasets, sharing a low number of sequences with them, and the saltern datasets were more similar to each other, making it possible to recognize the impact of a salinity gradient. Unexpectedly however, the community from IC21 was closer in structure to both the datasets, SS19 and SS33, although the salt concentration in IC21 was nearer to the dataset SS19, suggesting community composition is affected by local environmental characteristics. Thus, we focused this study on the intermediate salinity ponds and analyzed the datasets qualitatively and quantitatively to elucidate potential causes that might produce the observed differences.

**Figure 2:**
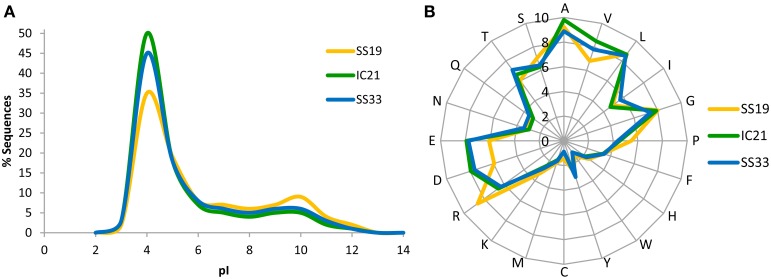
**Isoelectric point (pI) profiles (A) and amino acid frequency (B) of the predicted proteins in the metagenomic datasets SS19, IC21, and SS33**. The pI was computed for each translated read and is shown as a percentage of the dataset in intervals of bin width 2.

A well-known adaptive feature for living at high salt concentration is the enrichment of acidic amino acids in proteins allowing them to properly function at high cytoplasmic salinities (Soppa, 2006). Therefore, we analyzed the isoelectric point and amino acids frequencies of proteins, which would reflect this adaptation. Surprisingly, IC21 proteins were more similar to those in SS33 than to SS19 (Figure 2). The dataset IC21 showed an increase in acidic amino acids compared to SS19 that therefore indicates a greater presence of microorganisms using the “salt-in” strategy to osmotically balance their cytoplasm with their environment (Oren, 2008, 2013).

**Figure 3:**
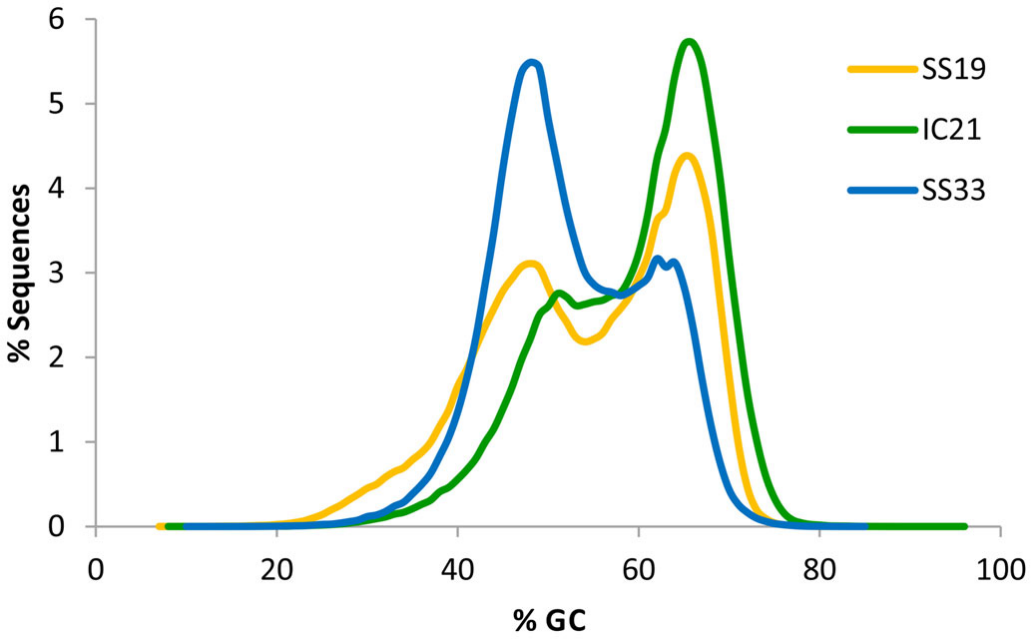
**Comparison of G+C% of sequences from metagenomic datasets SS19, IC21, and SS33**. G+C% was computed for each read and the percentage of the dataset in intervals of bin width 10 is shown.

High G+C content is often associated with the presence of haloarchaea, except for the well-known exception *Haloquadratum* and is a useful predictor of community composition. Analysis showed that the G+C content of IC21 (Figure 3) had a bimodal distribution more similar to SS19 than to SS33, and the predominant peak in both is at ~65%. This peak is consistent with the high G+C content associated with most halophilic archaea and bacteria described so far (Paul et al., 2008). Ponds from Santa Pola saltern (SS19 and SS33) exhibited a low G+C peak at 47.9%, which has previously been observed to increase at higher salinities (Ghai et al., 2011). This low G+C peak comes from *Haloquadratum walsbyi* (Bolhuis et al., 2006) and perhaps from the newly reported nanohaloarchaea (Ghai et al., 2011). In IC21 the low G+C peak is shifted to a value slightly higher than that observed in metagenomic datasets from Santa Pola ponds, SS19 and SS33, around 51–52%. This low G+C peak might correspond to genera containing representatives of halophilic bacterial genera such as *Halomonas*, *Salimicrobium*, or *Salinicoccus*. These genera have DNA G+C contents in the range of 52.0–74.3, 44.9–51.5, and 46–51.2%, respectively (de la Haba et al., 2011). Another possibility is that it corresponds to halophilic or halotolerant microorganism not yet described, or to AT-rich regions in haloarchaeal genomes (Ram Mohan et al., 2014) In members of the order *Halobacteriales* a “minor component” of the DNA (10–30% of the total DNA) with a G+C range of 51–59 mol% has been reported (Grant et al., 2001).

### Prokaryotic community structure

**Figure 4:**
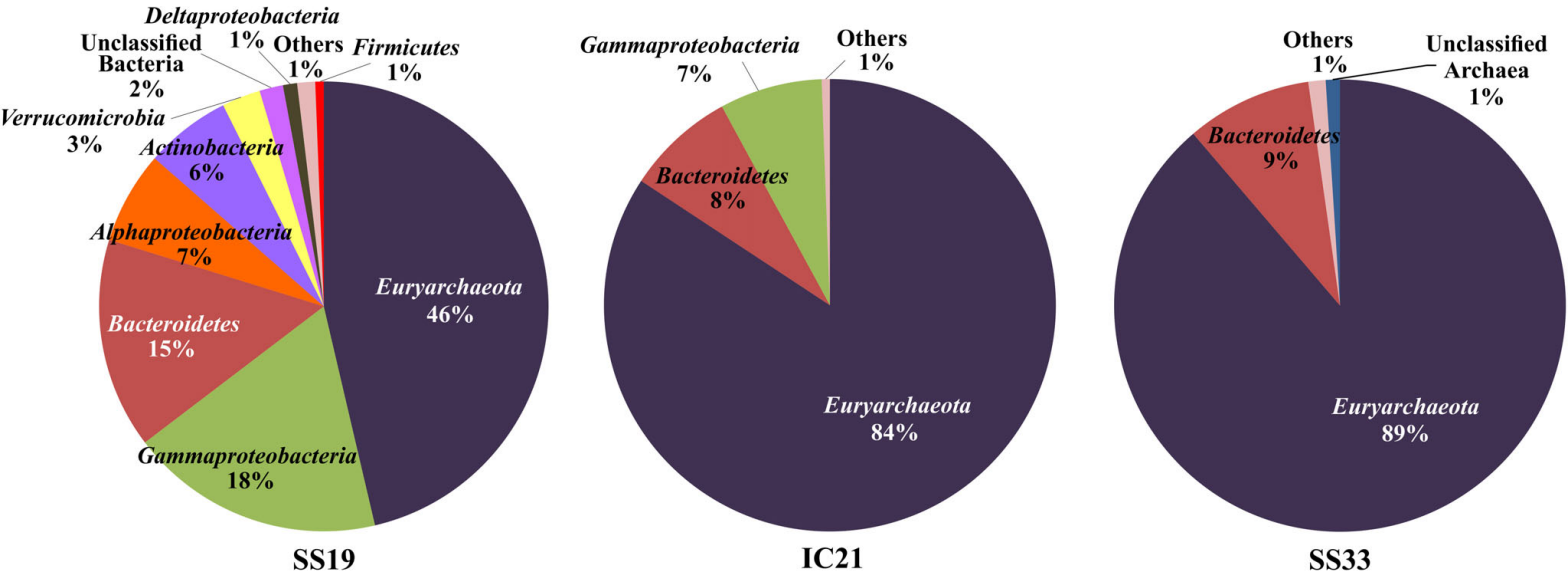
**Representation of high taxonomic levels affiliated to metagenomic rRNA reads**. Assigned sequences have an identity over 80% and a minimum length of 100 bp.

The taxonomic diversity was analyzed carrying out a search of the metagenomic sequences related to the 16S rRNA gene using the RDP database and selecting those sequences with a minimum length of 100 bp and an identity over 80% for higher taxonomic levels (Figure 4) and 95% for the genus level (Table 2).

**Table 2:** **Microbial diversity at genus level in the metagenomic datasets SS19, IC21, and SS33**.

**Sample**	**SS19**		**IC21**		**SS33**	
	*Halorubrum*	12.5%	*Halorubrum*	65.8%	*Haloquadratum*	29.5%
	*Haloquadratum*	7.9%	*Psychroflexus*	4.6%	*Halorubrum*	23.1%
	*Salinibacter*	6.4%	*Natronomonas*	3.2%	*Natronomonas*	5.7%
	*Natronomonas*	5.6%	*Haloquadratum*	1.9%	*Salinibacter*	4.7%
	“*Spiribacter*”	4.4%	“*Spiribacter*”	1.1%	*Haloplanus*	3.4%
	Haloredivivus^*^	3.2%				
	*Puniceicoccus*	1.9%				
	*Arhodomonas*	1.8%				
	Aquiluna^*^	1.5%				
	*Oceanicola*	1.1%				
Total number of sequences		1477		1538		888
Total number of genera		69		28		16

Assigned sequences have a 16S rRNA identity over 95% and a minimum length of 100 bp. Only those genera with more than 1% of assigned sequences are shown. SS19: Santa Pola saltern (19% salinity), IC21: Isla Cristina saltern (21% salinity), and SS33: Santa Pola saltern (33% salinity).

* Candidatus.

In Figure 4 it is observed that the bacterial community decreases sharply in IC21 compared to SS19, shifting to a largely archaeal community. Also identified are members of 14 higher taxa in SS19 but interestingly we found only six in IC21 and five in SS33. The phyla *Euryarchaeota* and *Bacteroidetes* and the class *Gammaproteobacteria* are shared by the three datasets, but there are numerically more sequences related to the phylum *Euryarchaeota* with increasing salinity and a concurrent decrease for the other two. Further, these data show that the phyla biodiversity in IC21 is more similar to SS33 than to SS19. Ghai et al. (2011) analyzed the changes of the biodiversity along a salinity gradient in two metagenomic datasets (with 19 and 37% salts) from Santa Pola saltern. The biodiversity detected in the crystallizer pond (SS37) is quite similar to that determined for SS33, due to their extreme salinities; however in the crystallizer pond only two phyla were found, corresponding to *Euryarchaeota* and *Bacteroidetes*.

The simplification of the prokaryotic community at higher salinities is also seen at the genus level: the number of sequences related to different genera decreases from 69 in SS19 to 28 and 16 in IC21 and SS33, respectively. The most abundant sequences in all three datasets are related to the archaeal genera *Halorubrum*, *Haloquadratum*, and *Natronomonas*, recruiting more *Halorubrum* sequences in SS19 and IC21 (12.5 and 65.8%, respectively) and *Haloquadratum* in SS33 (29.5%) (Table 2). The genus *Salinibacter* (belonging to the *Bacteroidetes*) is the next taxon highly represented in Santa Pola datasets with 6.4 and 4.7% of the sequences in SS19 and SS33, respectively. However, in IC21 the second predominant genus is *Psychroflexus*, also a member of the phylum *Bacteroidetes*, at 4.6% of the sequences. The six species comprising the genus *Psychroflexus* (*P. gondwanense*, *P. halocasei*, *P. salinarum*, *P. sediminis*, *P. torquis*, and *P. tropicus*) have been characterized as slightly or moderately halophilic bacteria and most of them have been isolated from saline environments (Bowman et al., 1998; Donachie et al., 2004; Chen et al., 2009; Yoon et al., 2009; Seiler et al., 2012). With respect to genera of the class *Gammaproteobacteria*, in SS19 and IC21, *Spiribacter* is identified as one of the most abundant. In spite of the strain “*Spiribacter salinus*” M19–40 being isolated from Isla Cristina saltern (Leon et al., 2013; López-Pérez et al., 2013), it was more abundant in SS19 compared to IC21 (4.4% vs. 1.1%, respectively). In fact, Ghai et al. (2011) reported a great abundance of sequences belonging to *Gammaproteobacteria* related to the genus *Alkalilimnicola*, which later was assigned to “*S. salinus*” M19–40 (López-Pérez et al., 2013). Although at the phylum or class level IC21 is more similar to SS33 at the genus level the Isla Cristina dataset differs in biodiversity and abundance from both Santa Pola datasets, demonstrating an overrepresentation of the genus *Halorubrum* and decrease in the sequences (0.4%) related to *Salinibacter* in IC21. Ghai et al. (2011) observed that in Santa Pola datasets (SS19 and SS37) the species *Salinibacter ruber* appeared as an abundant microorganism, but in IC21 it is much lower.

Traditional culture methods carried out in Santa Pola saltern determined that in intermediate salinity ponds there were a variety of moderately halophilic microorganisms (Rodríguez-Valera et al., 1985). Subsequent studies performed by molecular techniques indicated that these prokaryotic representatives belong in the groups *Gammaproteobacteria*, *Bacteroidetes*, and *Halobacteriaceae* and in a pond with 22% total salts 16S rDNA sequences were related to the genera *Psychroflexus*, *Halorubrum*, and *Natronomonas* (Benlloch et al., 2002); similar results were obtained in the dataset IC21. In a recent study of a pond from the Exportadora de Sal (ESSA) evaporative saltern in Guerrero Negro (Mexico) with 18% total salts a *Halorubrum*-like sequence nearly identical (>99.5% similar) to environmental sequences from the Santa Pola saltern as well as sequences 97% similar to “*S. salinus*” were reported (Dillon et al., 2013). Recently, Podell et al. (2013) found that the microbial composition from Lake Tyrrell (Australia) was correlated with concentrations of potassium, magnesium, and sulfate, but not sodium, chloride, or calcium ions. Sequences related to *Haloquadratum* were positively correlated with potassium, magnesium, and sulfate ions while sequences related to *Halorubrum*, *Haloarcula*, *Halonotius*, *Halobaculum*, and *Salinibacter* were negatively correlated with them. In addition, *H. walsbyi* shows a higher tolerance to Mg^2+^ than other halophilic archaea (Bolhuis et al., 2004; Burns et al., 2004). The differences found among Isla Cristina and Santa Pola datasets, mainly due to the dominance of sequences related to *Halorubrum* in IC21 and to *Haloquadratum* in SS19 and SS33, might be explained by the difference in the ionic composition of both samples as previously reported in other hypersaline habitats (Pagaling et al., 2009; Grant et al., 2011; Boujelben et al., 2012; Podell et al., 2013).

### Contigs of concentrator ponds

**Figure 5:**
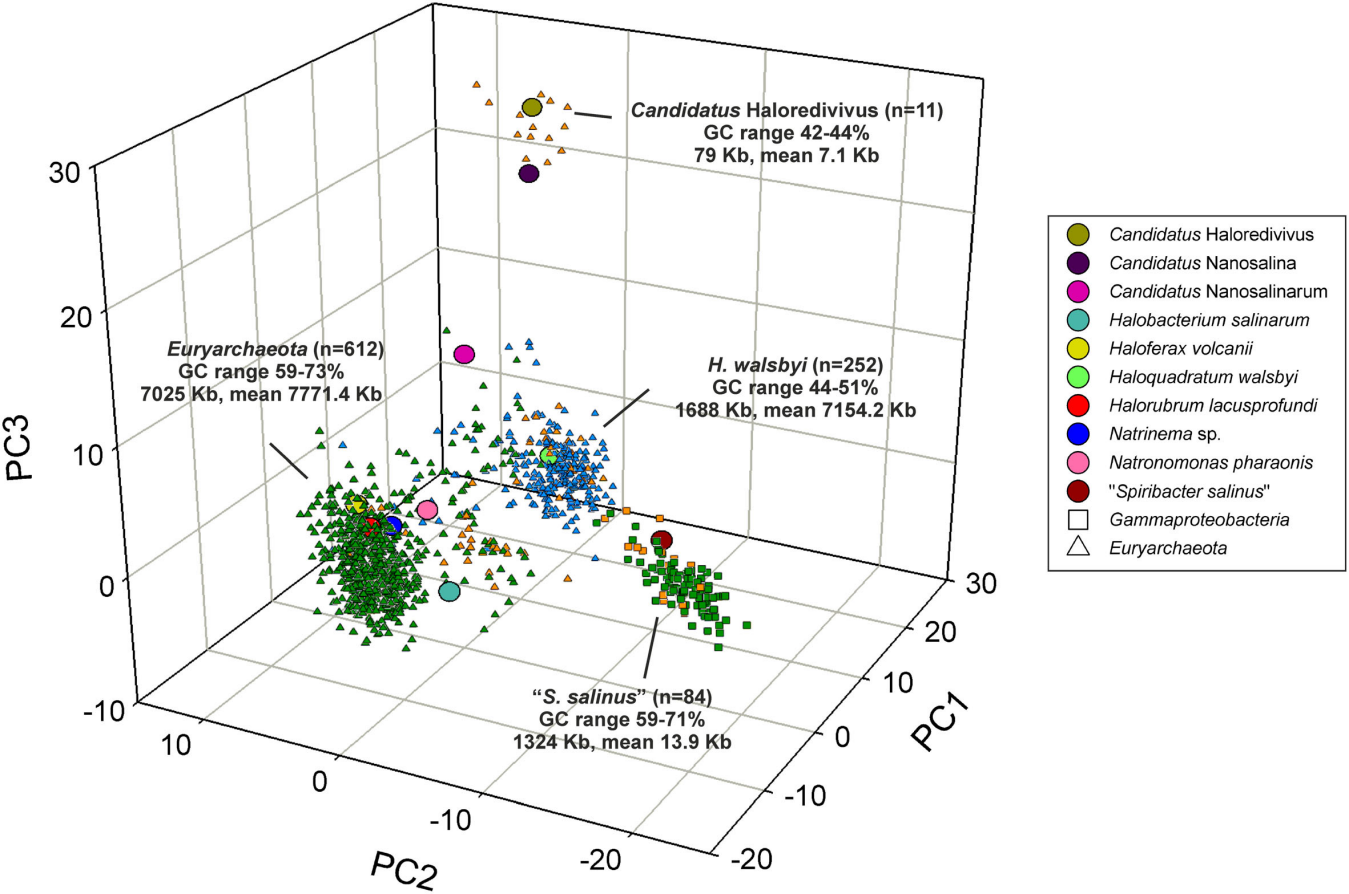
**Principal component analysis of tetranucleotide frequencies of assembled contigs from SS19, IC21, and SS33 datasets**. Reference genomes are shown as larger circles. The following types of contigs are shown: orange: SS19 contigs, Green: IC21 contigs, Blue: SS33 contigs, Square: Gammaproteobacterial contigs, Triangle: *Euryarchaeota* contigs. The total number of contigs for each cluster, the total length, mean length, and G+C% range is also indicated.

It is expected that contigs assembled from metagenomic reads will yield genomic fragments derived from the most abundant organisms in the sample (Ghai et al., 2011). Assembled contigs from the metagenomic datasets SS19, IC21, and SS33 were assembled and tested against the genome of an abundant organism in the datasets, *H. walsbyi*. In SS19, a total of 84 contigs larger than 5 kb were assembled, 69 could be assigned to the phylum *Euryarchaeota* and 15 to the class *Gammaproteobacteria* (Ghai et al., 2011). In IC21 710 contigs with at least 5 kb were obtained; 618 were assigned to the phylum *Euryarchaeota*, 85 contigs to the class *Gammaproteobacteria* and 7 contigs to viruses. With respect to SS33 a total of 248 contigs were assembled, 247 were assigned to *Euryarchaeota* and 1 to the phylum *Bacteroidetes*. A PCA on the normalized tetranucleotide frequencies of the contigs belonging to the most abundant groups was carried out (Figure 5). The contigs from SS19 and IC21 had consistent hits to taxa within the *Euryarchaeota* and *Gammaproteobacteria* and from SS33 the majority were *Euryarchaeota.* Contigs related to the phylum *Euryarchaeota* are grouped in two different clusters with low G+C content, one of them closely related to *H. walsbyi*, comprising 30 and 222 contigs of SS19 and SS33, respectively, and the second cluster related to the nanohaloarchaeon “*Candidatus* Haloredivivus” forming 11 contigs of the SS19. Additionally, we observed a third cluster including *Euryarchaeota* of high G+C content, comprising contigs from the three datasets that were related to genomes of extremely halophilic archaea. On the other hand, many contigs related to *Euryarchaeota* from the three datasets did not cluster with contigs related to *H. waslbyi* nor *Euryarchaeota* of high G+C content. Their G+C content values are between the extremely halophilic archaeal reference genomes and *H. walsbyi*; possibly these contigs belong to unknown hyperhalophilic archaea. A fourth cluster of *Gammaproteobacteria* contigs from SS19 (13 contigs) and IC21 (71 contigs) was closely related to the genome of “*Spiribacter salinus*” M19–40 (López-Pérez et al., 2013).

Because of the unique G+C content, the contigs with a 51–52% G+C in IC21 were further examined (Figure 3). Contigs associated with *Gammaproteobacteria* and viruses did not have this G+C content, ruling them out as contributors. However, among the nine contigs related to *Euryarchaeota*, three could clearly be assigned to *Halorubrum* and also had similar G+C content. These contigs mainly contain genes for ABC transporters, metallophosphoesterase, multi-sensor signal transduction histidine kinase, and hypothetical proteins.

**Figure 6:**
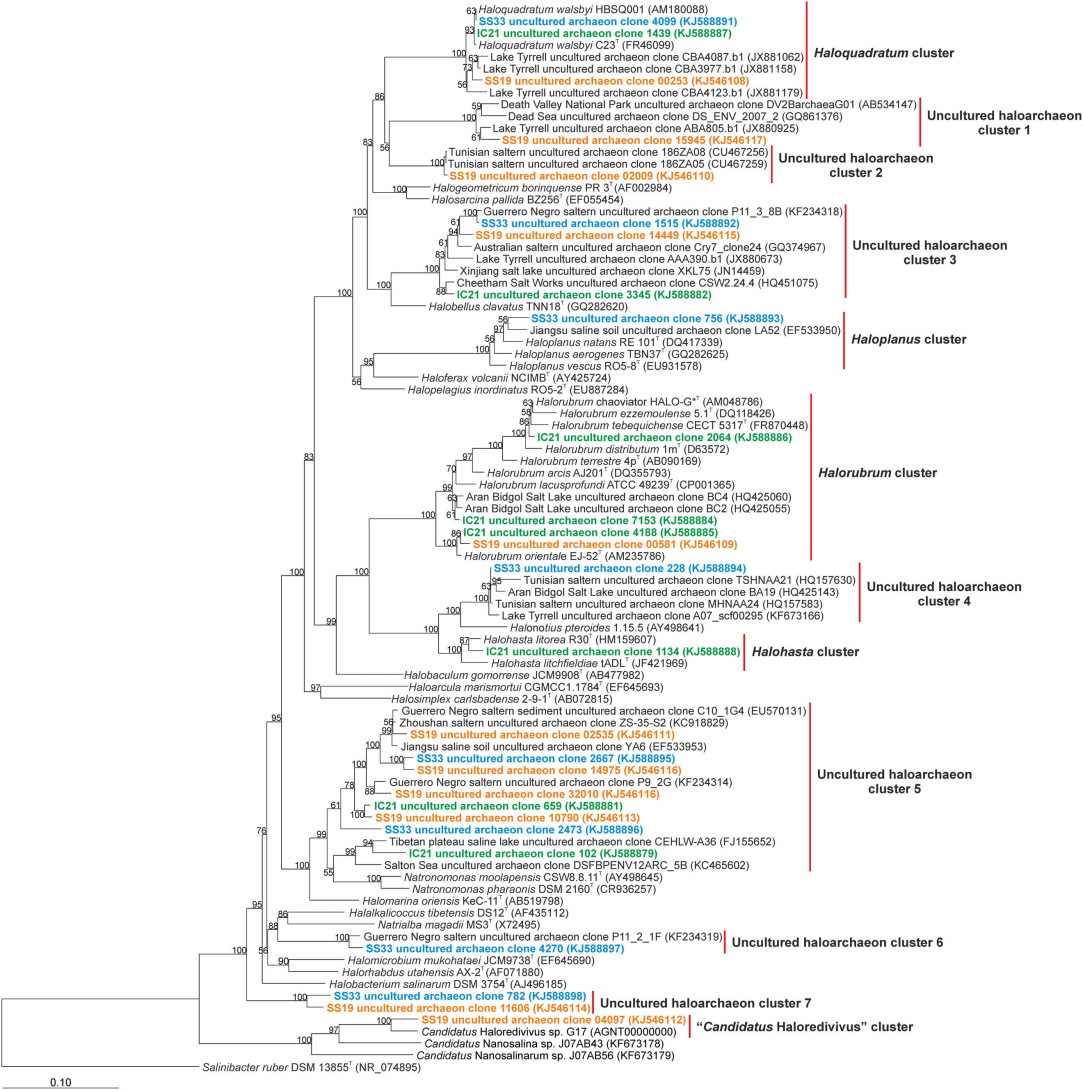
**Phylogenetic affiliation of the *Euryarchaeaota* 16S rRNA reads of the datasets SS19, IC21, and SS33**. Phylogenetic reconstruction was conducted by maximum likelihood (RAxML) with near full length (1300 nt) reference 16S rRNA gene sequences from a manually curated alignment and highly variable positions masked. Metagenomic reads were added without altering tree topology using maximum parsimony criterion in the ARB software package. Bootstrap values are indicated above nodes support ≥50%. The scale bar represents 10 base substitutions per 100 nt positions.

Further taxonomic analysis was performed on 16S rRNA genes from assembled metagenomic reads longer than 500 bp. We found eight 16S rRNA sequences from each of the datasets SS33 and IC21, and 11 sequences from the dataset SS19 that were analyzed using BLAST (Figure 6). One assembled sequence from each of the datasets grouped within the *Haloquadratum* cluster together with uncultured archaeal sequences from Lake Tyrrell, VIC, Australia (with 29% salinity). The 16S rRNA sequences from SS19, IC21, and SS33 had high similarity with *H. walsbyi* HBSQ001 of 100, 99.9, and 98.9%, respectively. Only one sequence from SS33 was found within the *Haloplanus* cluster with a similarity of 98.0% to *Haloplanus natans* and 97.8% to an uncultured archaeon sequence of a saline soil from Jiangsu (China). Three sequences from IC21 and one sequence from SS19 were included into the *Halorubrum* cluster. One sequence from IC21 shared with *Hrr. chaoviator* a similarity of 99.2%, another sequence from IC21 had a similarity of 99.4 and 99.3% with two different sequences of uncultured archaea from Aran-Bidgol salt lake (salinity over 30%) and other sequence from IC21 shared a similarity of 99.5% with a sequence from SS19 and with *Hrr. orientale*. Within the *Halohasta* cluster one sequence from IC21 had a similarity of 98.8% to *Halohasta litorea*. The “*Candidatus* Haloredivivus” cluster was represented by one sequence from SS19. The rest of the contigs could not be classified into known-archaeal genera. The assembled 16S rRNA sequences from the three datasets showing the presence of potential microorganisms related to *Haloquadratum* (although in IC21 representatives of this genus do not appear to be very abundant) and uncultured archaea in clusters 3 and 5. In IC21 the assembled sequences show a high abundance of members of the genus *Halorubrum*. A pattern regarding the community composition among the datasets studied appears to be absent, but we do find evidence that some sequences from different hypersaline environments are related to them. This suggests unknown environmental parameters affect community composition, and/or perhaps random dispersal and horizontal gene transfer of key adaptive genes play a role. For instance, Parnell et al. (2010) demonstrated that genes providing adaptation to their niche, rather than the taxa living there, structured halophilic communities in the Great Salt Lake.

**Figure 7:**
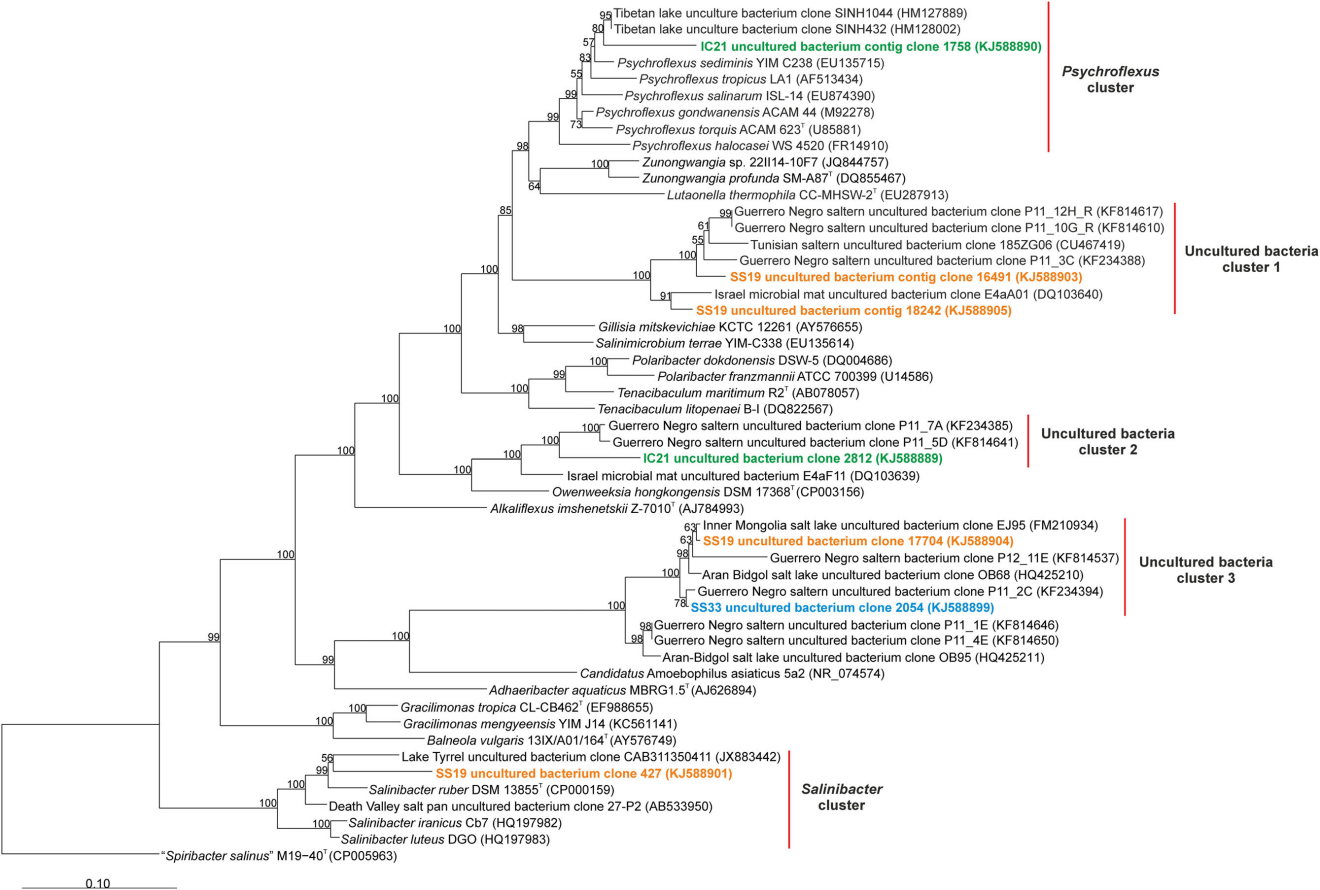
**Phylogenetic affiliation of the *Bacteroidetes* 16S rRNA reads of the datasets SS19, IC21 and SS33**. Phylogenetic reconstruction was conducted by maximum likelihood (RAxML) with near full length (1300 nt) reference 16S rRNA gene sequences from a manually curated alignment and highly variable positions masked. Metagenomic reads were added without altering tree topology using maximum parsimony criterion in the ARB software package. Bootstrap values are indicated above nodes support ≥50%. The scale bar represents 10 base substitutions per 100 nt positions.

Additionally, we found 16S rRNA assembled metagenomic reads longer than 500 bp related to the phylum *Bacteroidetes*: four from SS19, two from IC21 and one from SS33 (Figure 7). We used BLAST to search in the nr/nt database the sequences showing a higher similarity to the 16S rRNA assembled sequences of *Bacteroidetes*. A first cluster related to *Psychroflexus* was detected including one sequence from IC21. This sequence was similar to an uncultured bacterium from a Tibetan hypersaline lake (96.4%) and *Psychroflexus sediminis* (95.7%). One sequence from SS19 was within the *Salinibacter* cluster, showing a 92.1% similarity to *Salinibacter ruber*. The rest of the sequences from SS19, IC21, and SS33 could not be classified into any cluster containing cultured microorganisms. The 16S rRNA assembled metagenomic reads of *Bacteroidetes* show that in SS19 this taxon is more abundant than IC21 and SS33, with predominance of sequences related to uncultured bacteria, while in the dataset IC21 *Psychroflexus* was the genus that recruited more 16S rRNA sequences (Table 2). Neither of the 16S rRNA assembled sequences related to *Bacteroidetes* in IC21 are related to any of the assembled sequences of SS19 and SS33.

Although at high taxonomic levels IC21 is more similar to SS33 than SS19, the fact is that IC21 and SS33 are different with respect to the genera and their abundance observed as well as to the presence of different uncultured taxa. In saline and alkaline lakes in Iran a similar composition of microbial communities but differing in community structure has been reported (Makhdoumi-Kakhki et al., 2012).

### Metabolic profile

To analyze the metabolic diversity in the dataset IC21, we determined the relative abundance of individual genes involved in metabolic pathways of the datasets SS19, SS33, and IC21 (Supplementary Table 1) by searching predicted metagenomic proteins with the UniprotKB database (UniProt Consortium, 2014) using BLASTX (Camacho et al., 2008).

Metabolism based on energy from light was queried in our data sets. Because our screening protocol removed eukaryotic microorganisms, only prokaryotic microorganisms were considered in this analysis. The photosynthetic reaction centers, *psbA* and *psbD* genes, were not detected in the three datasets analyzed and therefore we can conclude that photosynthesis by prokaryotes was absent in these datasets. Instead, genes coding different types of rhodopsins were found suggesting that light is widely used as an energy source in these conditions, just not for carbon fixation. It was observed that bacteriorhodopsins and halorhodopsins increased in frequency at higher salinities. Bacteriorhodopsins associated with *Haloquadratum* were in a greater proportion in Santa Pola saltern datasets while in IC21 they were associated with *Halobacterium* and *Halorubrum*. Halorhodopsins related to *Halobacterium* were found in high proportion in all datasets but the number of sequences belonging to *Haloquadratum* is greater in Santa Pola saltern and those from *Halorubrum* are higher in IC21. Sequences related to bacteriorhodopsins and halorhodopsins show clearly the abundance of *Haloquadratum* in Santa Pola SS19 and SS37 datasets and *Halorubrum* in Isla Cristina IC21 dataset.

Microorganisms under osmotic stress conditions use different survival strategies. Most bacteria maintain cell integrity through accumulation of compatible solutes (“salt-out” strategy). Sequences related to genes of compatible solutes were mainly glutamate synthase, betaine transporters, glycerol kinase, and glycerol-3-phosphate dehydrogenase, and at lower frequencies were glycerol and glutamate transporters. In IC21 lower number of sequences related to choline dehydrogenase, glutamate synthase, and trehalose synthase were observed compared to Santa Pola datasets, suggesting a higher synthesis of these compatible solutes in Santa Pola saltern. The proportion of sequences related to compatible solutes decreased with the salinity except for glycerol degradation (glycerol kinase, glycerol-3-phosphate dehydrogenase, and dihydroxyacetone kinase), increasing the number of sequences in the dataset SS33 compared to SS19 (number of sequences relative to the total number of metagenomic sequences). This result could indicate a higher presence of the algae *Dunaliella* or a higher primary production of glycerol, which is the predominant compatible solute in *Dunaliella* or alternatively, the oxidation of glycerol to dihydroxyacetone, that has been studied in the species *Salinibacter ruber* and is used as a growth substrate by *H. walsbyi* and *Haloferax volcanii* (Elevi Bardavid and Oren, 2008; Ouellette et al., 2013). Therefore, glycerol and probably dihydroxyacetone are considered as the main carbon source and energy for the heterotrophic community in salterns (Borowitzka and Brown, 1974; Borowitzka et al., 1977; Ouellette et al., 2013).

With respect to the nitrogen cycle, it seemed to be simplified in the saltern datasets analyzed, with a decrease of the number of sequences involved in the reduction of nitrate to nitrite by nitrate reductase and nitrite to nitric oxide by nitrite reductase in SS33.

In salterns, sulfate is concentrated along the ponds until its saturation and precipitates forming calcium sulfate (gypsum) (Landry and Jaccard, 1984). Dissimilatory sulfate reduction has been reported until salt concentrations of 24% NaCl (Oren, 1988). We detected sequences of genes involved in a complete dissimilatory sulfate reduction (sulfate adenylyltransferase, adenylylsulfate kinase, phosphoadenylylsulfate reductase, and sulfite reductase) in all datasets studied, except for adenylylsulfate kinase in SS33 dataset. The microbial communities of salterns are traditionally considered heterotrophic, but chemolithotrophic bacteria can also be abundant and active in extreme conditions. In particular, chemolithotrophic sulfur-oxidizing bacteria are able to adapt well to hypersaline conditions because the complete oxidation of sulfide or thiosulfate to sulfate has high energy efficiency (Oren, 1999). In spite of this, we only found some sequences of sulfide dehydrogenase that oxidizes sulfide to sulfate in SS19. Therefore, an incomplete cycle of sulfate is observed in the datasets. In fact, salterns are generally considered eutrophic media and so the pressure of selection that favors organisms with biosynthetic capabilities with full paths is probably weak (Rodríguez-Valera et al., 1981). Additionally, this pathway may be carried out by phototrophic sulfur-oxidizing bacteria in anoxic sediments, as hydrogen sulfide is considered an important transporter of electrons between the aerobic and anaerobic habitats (Jørgensen, 1982).

Phosphate regulon (Pho) plays a key role in phosphate homeostasis, products are involved in the transport and use of several forms of phosphates (Torriani and Ludtke, 1985; Shinagawa et al., 1987; Wanner, 1987, 1993). In the datasets studied a scarce number of sequences related to genes included in Pho regulon, as *phoR* (environmental phosphate sensor) and *phoB* (regulon activator) were found. However, in the dataset IC21 more sequences corresponding to the negative regulator protein of Pho regulon, PhoU and less for genes involved in the utilization of phosphonate were detected compared to the datasets SS19 and SS33. In Santa Pola saltern the total phosphorus concentration increases with the salinity (Rodríguez-Valera et al., 1985). However, the brines support high Mg^2+^ concentrations limiting the availability of inorganic phosphate (Bolhuis et al., 2006) and induce the use of phosphonate (Fox and Mendz, 2006). A recent study suggests the utilization of DNA as a phosphate source (Chimileski et al., 2014).

Overall, the main differences found among Isla Cristina and Santa Pola datasets at the metabolic level were a higher number of sequences related to genes involved in the synthesis of compatible solutes (such as choline dehydrogenase, glutamate synthase, and trehalose synthase) and in the utilization of phosphonate in Santa Pola datasets with respect to IC21. This is related to the microbial strategies of haloadaptation to these extreme environments by the different microbial communities present of these habitats as well as the ionic composition of the samples.

## Conclusions

Santa Pola saltern was built in 1890 over an ancient freshwater lake and close to the Mediterranean Sea (Dulau, 1983). By contrast, Isla Cristina saltern was built in 1955 over wetlands at the marsh of the river Carreras in the village of Isla Cristina, closely located to food-processing industries (Moreno et al., 2009). Santa Pola saltern is subjected to climatic conditions characterized by low annual rainfall and moderate temperatures, with little fluctuation between summer and winter (Rodríguez-Valera et al., 1985). However, Isla Cristina saltern is subjected to rainfall seasons, high solar radiation, and larger temperature fluctuations between day and night (Moreno et al., 2009). Although salinity has been considered the main factor involved in the structure of microbial biodiversity in saline aquatic systems, the data from our study strongly suggests that other factors may influence the composition of hypersaline aquatic microbial communities such as geographic locations (Naor et al., 2012; Zhaxybayeva et al., 2013) and environmental characteristics. The environmental conditions in Santa Pola saltern are more stable than in Isla Cristina saltern, therefore, the prokaryotic community in Santa Pola saltern is more stable over time, and in Isla Cristina saltern the habitat might be continuously recolonizing. Therefore, the differences between these two environments might be due to stable or unstable environmental conditions. The IC21 dataset looks more similar to SS33 dataset because they are mainly composed of representatives of the phylum *Euryarchaeota*, but the reality is that the community structure in IC21 is different because the most abundant genus in IC21 is *Halorubrum*, in contrast to *Haloquadratrum*, which predominates in SS33 dataset. The phylum *Bacteroidetes* is present in all datasets, but in the Santa Pola saltern datasets the most abundant genus of this phylum is *Salinibacter*, while in Isla Cristina saltern dataset is the genus *Psychroflexus*. Additionally, in Santa Pola datasets there are a higher number of sequences related to genes involved in the synthesis of compatible solutes and in the utilization of phosphonate, indicating some differences in the functional activity. Despite the results obtained, it is not clear what is causing the variations between these salterns; a detailed physico-chemical comparative study would be required to elucidate if the microbial structure is being influence by abiotic factors or by biogeographic situation.

## Author contributions

Antonio Ventosa, R. Thane Papke, Francisco Rodriguez-Valera, and Ana B. Fernández conceived the study. Ana B. Fernández and Cristina Sánchez-Porro obtained the metagenome. Ana B. Fernández, Blanca Vera-Gargallo, and Rohit Ghai performed the analysis. Ana B. Fernández, R. Thane Papke, Francisco Rodriguez-Valera, and Antonio Ventosa wrote the manuscript. All authors read and approved the final manuscript.

### Conflict of interest statement

The authors declare that the research was conducted in the absence of any commercial or financial relationships that could be construed as a potential conflict of interest.
