# Supplementary material for: Comparison of prokaryotic community structure from Mediterranean and Atlantic saltern concentrator ponds by a metagenomic approach
Source: Front Microbiol. 2014 May 8;5:196. doi: 10.3389/fmicb.2014.00196 (PMC4021199; doi:10.3389/fmicb.2014.00196)
Supplement: Supplementary file 1 [file DataSheet1.DOCX]

**Supplementary Table 1 | Number of sequences of genes in each dataset.** SS19: Santa Pola saltern (19 % salinity), IC21: Isla Cristina saltern (21 % salinity) and SS33: Santa Pola saltern (33 % salinity). Assigned sequences have an identity over 70 % and a minimum length of 100 bp.

|  | |  | **SS19** | **IC21** | **SS33** |
| --- | --- | --- | --- | --- | --- |
| **Rhodopsins** | Bacteriorhodopsin | | 164 | 162 | 195 |
|  | Halorhodopsin | | 119 | 188 | 156 |
|  | Sensory Rhodopsin | | 3 | 20 | 10 |
|  | Xanthorhodopsin | | 60 | 0 | 13 |
|  | Xenorhodopsin | | 0 | 0 | 0 |
|  | Proteorhodopsin | | 3 | 1 | 0 |
| **Compatible solutes** | Betaine (glycine betaine aldehyde dehydrogenase) | | 18 | 3 | 0 |
|  | Betaine (choline dehydrogenase) | | 113 | 1 | 120 |
|  | Betaine transporters | | 738 | 65 | 34 |
|  | Ectoine (ectoine synthase) | | 60 | 14 | 0 |
|  | Hidroxiectoine (ectoine hydroxylase) | | 0 | 0 | 0 |
|  | Ectoine transporters | | 73 | 28 | 0 |
|  | Glutamate (glutamate synthase) | | 1134 | 270 | 659 |
|  | Glutamate (glutaminase) | | 2 | 5 | 0 |
|  | Glutamate transporters | | 277 | 276 | 176 |
|  | Glycerol degradation I (Glycerol kinase) | | 398 | 510 | 303 |
|  | Glycerol degradation I (Glycerol-3-phosphate dehydrogenase) | | 570 | 317 | 622 |
|  | Glycerol degradation II (Glycerol dehydrogenase) | | 0 | 0 | 0 |
|  | Glycerol degradation II (glycerol phosphatase) | | 0 | 0 | 0 |
|  | Glycerol transporters | | 296 | 44 | 14 |
|  | Trehalose (trehalose 6-phosphate phosphatase) | | 24 | 3 | 0 |
|  | Trehalose (trehalose phosphorylase) | | 29 | 2 | 0 |
|  | Trehalose (trehalose 6-phosphate synthase) | | 41 | 7 | 7 |
|  | Trehalose (trehalose synthase) | | 87 | 8 | 32 |
|  | Trehalose transporters | | 38 | 11 | 8 |
| **N cycle** | Nitrate reductase | | 20 | 26 | 5 |
|  | Nitrite reductase | | 14 | 47 | 6 |
|  | Nitric oxide reductase | | 26 | 3 | 0 |
|  | Nitrous oxide reductase | | 15 | 4 | 2 |
|  | Nitrogenase | | 0 | 0 | 0 |
|  | Ammonia monooxygenase | | 0 | 0 | 0 |
| **S cycle** | Sulfate adenylyltransferase | | 599 | 382 | 309 |
|  | Adenylylsulfate kinase | | 394 | 96 | 0 |
|  | Phosphoadenylylsufate reductase | | 383 | 560 | 250 |
|  | Adenylylsulfate reductase | | 0 | 0 | 0 |
|  | Sulfite reductase | | 175 | 332 | 367 |
|  | Sulfide dehydrogenase | | 19 | 0 | 0 |
| **P cycle** | PhoR (histidine kinase) | | 84 | 8 | 33 |
|  | PhoB (response regulator) | | 48 | 17 | 0 |
|  | PhoU (Phosphate transport system regulatory protein) | | 137 | 719 | 233 |
|  | Phosphate transporters | | 926 | 1106 | 1153 |
|  | Phosphonate utilization | | 701 | 441 | 767 |
| **Total number of metagenomic sequences** | | | **1315367** | **1223923** | **842872** |
